# Investigating effects of divided attention at test on memory accessibility and precision in a continuous report paradigm

**DOI:** 10.3758/s13421-026-01868-4

**Published:** 2026-04-17

**Authors:** Nursima Ünver, Eren Günseli

**Affiliations:** 1 Department of Psychology, Sabanci University, Orta Mahalle, 34956 Istanbul, Turkey; 2 University of Toronto, Toronto, ON Canada; 3 Ernst Strüngmann Institute (ESI) for Neuroscience in Cooperation with Max Planck Society, Deutschordenstraße 46, 60528 Frankfurt am Main, Germany

**Keywords:** Mixture modeling, Multi-tasking, Distraction, Episodic memory

## Abstract

Previously reported tests for effects of dividing attention during long-term memory (LTM) retrieval have yielded inconclusive findings. This may be due to most prior work relying on categorical recall or recognition measures, which lack sufficient precision to detect subtle effects. Here, we provide the first test of divided attention effects on LTM retrieval using a continuous report paradigm, allowing retrieval errors to be measured with greater precision. In Experiment 1, participants (*N* = 44) encoded object–orientation associations and later retrieved object orientations under either full or divided attention while performing a visual search task. On each trial, error was computed as the difference between the reported and studied orientations of the tested object. Dividing attention during retrieval resulted in increased errors. To characterize the nature of these errors, we analyzed error distributions using both a standard mixture model (SMM) and the Target Confusability Competition (TCC) model. The SMM showed that divided attention reduced memory accessibility, while the precision of retrieved memories remained unaffected. The TCC model provided better fits and similarly indicated reduced memory strength under divided attention. Experiment 1 included an additional working memory load due to withholding the divided attention task response throughout the main retrieval task; Experiment 2 (*N* = 44) replicated the main findings while eliminating this load by having participants respond to the secondary task before the retrieval response. Together, these findings show that continuous report measures reveal costs of divided attention on LTM retrieval that primarily reflect reduced retrieval success or strength rather than reduced representational precision.

## Introduction

Imagine trying to recall the appearance of a cherished childhood toy. Such memories are stored in long-term memory (LTM) (Anderson, 1983; Cowan, 2001; Foster & Awh, 2019; Oberauer, 2002). If you attempt to remember this toy while simultaneously engaging in a conversation with a friend, you might struggle to conjure up the mental image of the toy. This difficulty arises because dividing attention across tasks can impair our ability to access our memories (Craik et al., 2018; Dodson et al., 1998; Fernandes & Moscovitch, 2002, 2003; Troyer et al., 1999). Understanding the impact of divided attention on retrieving LTMs is crucial, given the significance of memories and memory-driven behavior in human everyday functioning and well-being (Jetten et al., 2010; Schacter, 2013; Schacter et al., 2015; Sutin et al., 2021).

## Effects of divided attention on memory retrieval

Early research on the effects of divided attention on LTM retrieval suggested that, unlike encoding, retrieval processes operate relatively automatically. For example, Baddeley et al. (1984) and Craik et al. (1996) found that performing a secondary task during retrieval of verbal material produced little to no impairment in retrieval performance. One factor that modulates the effect of divided attention on retrieval is the type of the retrieval task. Studies using recall often report divided attention costs (Craik et al., 2018; Fernandes & Moscovitch, 2002, 2003), while evidence for such costs in recognition tasks is mixed (Anderson et al., 1998; Baddeley et al., 1984; Greene et al., 2021; Gruppuso et al., 1997; Hicks & Marsh, 2000; Lozito & Mulligan, 2006). Moreover, retrieval tasks that require recollection tend to be affected more by divided attention than those that can be performed based on familiarity or less precise information (Dodson et al., 1998; Gruppuso et al., 1997). For example, in Dodson et al. (1998), where participants heard words spoken by different voices, a divided attention task disrupted the retrieval of specific source information (exact voice) but not partial source information (gender of the voice). These results suggest that the costs of divided attention are stronger for recall or the recollection of specific details compared to recognition or familiarity.

What makes the effects of divided attention more visible on a recall task compared to a recognition task? One possibility is that divided attention disrupts the retrieval mode, thus preventing the establishment of detailed recollection, but not the reactivation of sensory traces, which may be sufficient to generate a familiarity signal that enables recognition. This explanation is consistent with the component process model, which suggests that memory retrieval consists of two components: the reactivation of the stored memory trace and the initiation and maintenance of retrieval mode. Notably, Moscovitch (1992) proposed that memory trace reactivation occurs automatically by the hippocampus without requiring attention. On the other hand, Moscovitch and colleagues (Fernandes & Moscovitch, 2002, 2003; Moscovitch, 2008) proposed that recall tasks additionally require being in a retrieval mode until the reactivation of the memory trace, which is regulated by the prefrontal cortex (PFC) and requires attention. There is indirect support for this model, such as free-recall tasks being affected by damage or dysfunction to the PFC (Moscovitch, 1994). A similar distinction was also proposed by Jacoby (1991), who posited that divided attention during retrieval hampers recollection (an intentional process) but does not affect familiarity (an automatic process). However, to our knowledge, there is no direct evidence for an automatic component of memory retrieval that can suffice for accurate responses in recognition versus recall tasks.

Another factor that modulates the effect of divided attention on retrieval is the similarity between the representations used for retrieval and those used for divided attention. Visual divided attention tasks, such as card sorting or visual continuous reaction time (CRT), did not impair verbal free-recall accuracy (Baddeley et al., 1984; Craik et al., 1996). On the other hand, Fernandes and Moscovitch (2000) observed greater costs on a verbal memory retrieval task when attention was divided with a word-monitoring task compared to a digit-monitoring task. Therefore, divided attention may disrupt retrieval when the distractor task and retrieved information tap onto the same representational system (e.g., verbal-verbal). Given the overlap of neural networks that represent newly acquired and retrieved information, this effect could be due to increased interference in the neural mechanisms that represent information encoded for the divided attention task and information reinstated via LTM retrieval (Favila, Lee, & Kuhl, 2020; Fukuda & Woodman, 2017; Gisquet-Verrier & Ricci, 2012; Long et al., 2018). Thus, this material-specificity principle suggests that to reliably detect divided attention effects on retrieval, the secondary task must engage representational resources similar to those required for the memory content.

## Dissociating accessibility, precision, and memory strength in retrieval

Traditional studies have measured memory retrieval using binary outcomes (i.e., correct vs. incorrect). While informative, such measures cannot reveal whether retrieval failures reflect complete inaccessibility of the memory trace, degradation of memory precision, or confusion with similar competing memories.

Different computational frameworks make distinct assumptions about how memory errors arise. Mixture models (Bays et al., 2009; Harlow & Yonelinas, 2016; Zhang & Luck, 2008) propose that errors reflect two discrete states: on some trials, the memory is accessible and responses cluster around the target value with variable precision; on other trials, memory fails completely and participants guess randomly, forming a uniform response distribution. This framework treats memory success and precision as independent parameters (see *Analyses* section for a detailed explanation of our implementation of the mixture model). Originally developed for working memory using continuous features like color and orientation (Zhang & Luck, 2008), the mixture-modeling approach has been increasingly applied to episodic LTM (Berens et al., 2020; Brady et al., 2013; Fan & Turk-Browne, 2013). A similar model has been developed by Harlow and Donaldson (2013) for episodic memory using word-location associations. They also dissociate memory success from precision, but use a Cauchy distribution (with heavier tails suitable for modeling spatial errors) rather than a von Mises distribution (Zhang & Luck, 2008) to characterize successful retrieval with variable precision. Harlow and Yonelinas (2016) extended this work by showing that participants can subjectively distinguish between these two aspects of episodic memory retrieval on a trial-by-trial basis, with confidence ratings predicting retrieval success while separate precision ratings reflected the fidelity of retrieved information.

Several factors selectively influence accessibility while preserving precision. For example, retrieval practice enhances the accessibility of representations while precision remains unchanged (Sutterer & Awh, 2016), and forgetting over time decreases accessibility while precision remains stable (Berens et al., 2020). These findings align with theoretical accounts suggesting that factors affecting retrieval cues and context (Godden & Baddeley, 1975; Tulving & Thomson, 1973) primarily influence accessibility by facilitating memory search. Conversely, other factors selectively affect precision while leaving accessibility relatively intact. Studies using continuous report paradigms found that the precision of items in working memory decreases with increasing memory load (Biderman et al., 2019; Zhang & Luck, 2008), duration of storage (Rademaker et al., 2018), and perceptual interference (van Moorselaar et al., 2015). Moreover, Klyszejko et al. (2014) found that when representations in working memory are prioritized by a reward or cue, recall precision was higher than when they were not cued, suggesting that the precision of representations in working memory is affected by attention. In LTM, Fan and Turk-Browne (2013) examined how retrieving a single feature (i.e., color) of an object affects the precision of other features. They found that when people retrieved one feature, the probability of guessing the second feature increased, while precision remained the same, demonstrating that accessibility and precision can be independently affected by retrieval.

At the neural level, accessibility and precision during episodic memory retrieval have been associated with BOLD (blood oxygenation-level dependent) activity in distinct brain regions – the hippocampus and angular gyrus, respectively (Richter et al., 2016). Similarly, Korkki et al. (2023) found that in older adults, age-related reductions in retrieval success were associated with decreased hippocampal activity, whereas reductions in memory precision were linked to diminished angular gyrus activity and reduced gray matter volume in this region. Critically, angular gyrus volume predicted memory precision even after controlling for retrieval success, demonstrating the anatomical specificity of precision deficits. Hou et al. (2025) also partially replicated these findings, showing that the angular gyrus was specifically sensitive to precision rather than accessibility, providing neural evidence that these parameters are dissociable features of memory retrieval. However, how the accessibility and precision parameters are affected by divided attention during episodic memory retrieval remains untested. Referring to our initial example, while a decrease in precision may exert itself as a loss in fidelity regarding the details of the toy, such as its color, texture, and specific features, a loss of accessibility due to divided attention might make it more challenging to retrieve the memory altogether.

An alternative framework, the Target Confusability Competition (TCC) model (Schurgin et al., 2020), attributes all errors to continuous competition between the target memory and similar representations, without positing discrete guessing states. In the TCC model, larger errors arise from stronger interference from perceptually similar non-target representations rather than from categorical memory failures. While these models differ in their theoretical assumptions about memory responses, both frameworks allow us to test whether divided attention affects retrieval success and/or strength.

## The present study

To examine how divided attention affects the accessibility and precision of LTMs, we used a continuous report paradigm in which participants reproduced the orientation of real-world object images on a continuous scale. This approach provided several benefits. First, it allowed using a mixture modeling approach on the continuous error distribution to estimate the accessibility of memories (i.e., whether participants report the target memory) and their precision (i.e., how precisely the target memory is reported in terms of its exact feature value). Second, the task involves associative memory (binding objects to specific orientations), which typically shows larger divided attention costs than item memory tasks (Dodson et al., 1998; Troyer et al., 1999; but see Greene et al., 2021). Finally, by pairing visual-spatial memory retrieval with visual-spatial secondary tasks, we created conditions in which the material-specificity principle predicts robust divided attention effects.

Previewing our key findings, divided attention during retrieval clearly impaired memory performance. Mixture modeling revealed that divided attention impaired only accessibility, not precision, of memories. Even though this suggests a dissociation between the accessibility and precision, model comparisons favored the unitary TCC model across all participants in both attention conditions. This challenges the assumption that accessibility and precision are independently affected, and suggests that divided attention may instead alter an underlying unitary memory strength signal. Taken together, this study sheds light on the role of attention and control processes in LTM retrieval.

## Experiment 1

### Method

#### Sampling plan

We used G*Power to calculate the target number of participants. There were no studies that tested the effects of divided attention on the accessibility and precision of LTM retrieval. Therefore, we assumed a medium effect size (Cohen’s *d* = 0.5) given the mixed findings of the prior studies that have found large effects (Craik et al., 2018; Fernandes & Moscovitch, 2003; Skinner & Fernandes, 2008) and no effect at all (Lozito & Mulligan, 2006; Weeks & Hasher, 2017). This calculation resulted in 44 participants with 90% power in a paired-sample *t*-test (alpha =.05).

#### Participants

Fifty-one undergraduate students participated in the experiment for course credit. Participants who reported having normal or corrected-to-normal vision and having no neuropsychological disorders were accepted for the experiment. Seven participants’ data were removed from the analysis: Four participants’ mean absolute error (e.g., the difference between the original and reported orientations) exceeded the chance level (90˚), and three participants’ parameter estimates were 2.5 standard deviations away from the grand average. Thus, the final analyses included 44 participants (28 female, 16 male; *M*_age_ = 21.16 years). The study was approved by the institutional ethics committee.

#### Materials

**Fig. 1:**
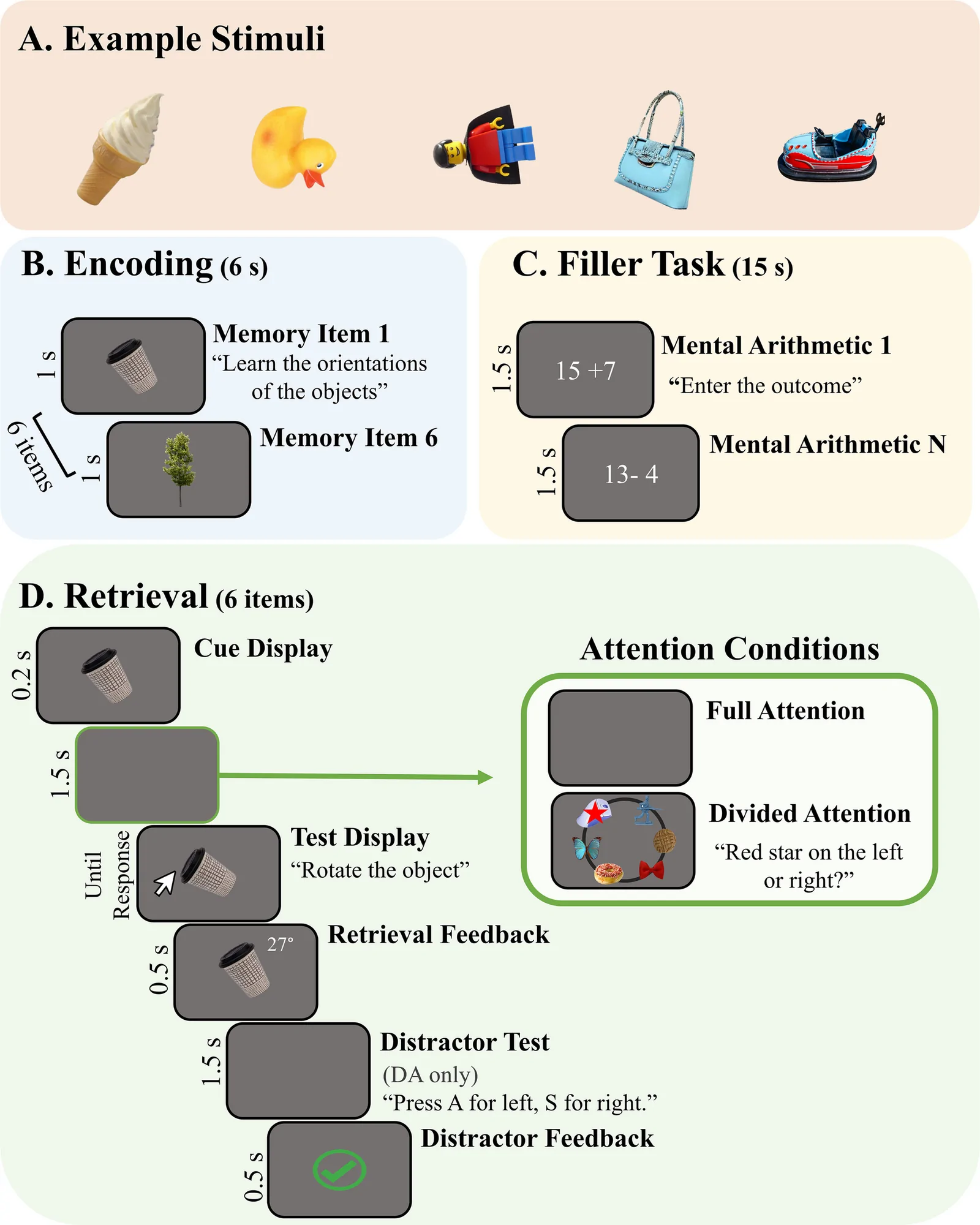
Illustration of the stimuli and the experimental procedure. The experimental procedure consisted of three main phases: encoding, filler task, and retrieval. (**A**) Each object image was presented in a randomly selected orientation. (**B**) During encoding, participants were presented with six objects with distinct angular orientations. (**C**) The filler task was to perform mental arithmetics for 15 s. (**D**) Right after the filler task, participants were given a retrieval cue followed by the test display that involved a probe object. Participants rotated the object to match the remembered orientation. The 1.5 s between the cue and the test contained a blank screen in the full attention condition, while it contained a visual search task in the divided attention condition (intermixed). Feedback was provided after each task. *Note:* In the actual divided attention task display, there were 12 objects; here, it is depicted as six for simplicity

The experiment was programmed and run through PsychoPy (Peirce et al., 2019). Stimuli were presented on a 1,920 × 1,080-pixel display at a viewing distance of approximately 70 cm. The stimulus set consists of 1,598 real-world object images with an approximate resolution of 250 × 250 pixels (Konkle et al., 2010; Konkle & Caramazza, 2013; Konkle & Oliva, 2012), 180 memory categories, and 20 distractor categories. A single object image was selected from each memory category for the memory task and randomly assigned to either full or divided attention conditions for each participant. We randomly sampled 180 unique orientations spanning the full 360° range (−179° to +180°), with values spaced roughly every 2° to create 180 different object-orientation associations for each participant to memorize (Fig. 1A). The size of each object image was approximately 11° × 11° of visual angle.

#### Design

The experiment has two within-subject conditions (full attention and divided attention) randomly intermixed across trials. Each block consisted of an encoding phase, a filler task phase, a distraction phase (except for the full attention condition), and a test phase. There were 60 blocks of six (three full attention, three divided attention) trials, totaling 360 trials. In the first half of the study, participants learned the 180 object-orientation associations. In the second half, participants restudied and were retested on the same 180 orientation associations under the same attention conditions. The second presentation was included to increase statistical power, ensuring 180 trials per attention condition for reliable mixture model estimation. Participants began the second presentation only after completing retrieval for all items in the first, so no item was repeated immediately, and the trial order was randomized within presentations. Participants were not informed that they were retested on the same objects. Data from both halves were included in the analyses.

#### Procedure

Before the experiment, participants completed computer-based consent and demographic forms. Then, they were given detailed instructions about the study and completed three practice blocks of 18 memory tests with the experimenter.

Each block began with an encoding phase in which six object images with different angular orientations were sequentially presented at the center of the screen, each for 1 s. Participants were instructed to learn these object-orientation associations. Next, they completed a 15-s filler task where they answered basic mathematical questions. After the filler task, a retrieval phase of six trials started. First, participants saw one of the objects at the center of the screen as a retrieval cue for 0.2 s. In the full attention condition, participants began the test phase after 1.5 s, where they needed to rotate the object with the mouse to match the memory orientation. Their response was saved with a mouse click. Lastly, they received feedback on the degree difference between the correct orientation and their response. In the divided attention condition, at the offset of the last retrieval cue, participants performed a 1.5-s visual search task. The search display contained 12 novel objects placed on a dark gray circle, and one of the objects had a red star on it. Participants’ task was to locate the red star, and later to report whether it was on the left or right side of the screen. While holding that information in mind, participants needed to respond to the retrieval task by rotating the object to match the memorized orientation, as in the full attention condition. After receiving the degree difference feedback, they were required to respond to the divided attention task by pressing “A” if the red star was on the left and “S” if it was on the right within 1.5 s. Lastly, they received visual feedback for the visual search task (“correct,” “incorrect,” or after 1.5 s “miss”). Participants were not given any instructions to prioritize one task over the other. Upon completing one block of six trials, participants were informed about their accuracy and were able to take a self-paced break.

### Analyses

#### Mixture modeling

In continuous report tasks, the distribution of recall error (i.e., the difference between the target orientation and the reported orientation) can be modeled as a combination of two distributions. One is a circular normal distribution (i.e., von Mises) where recall errors are expected to cluster around the target orientation with some degree of noise. The width of this cluster is represented by the precision parameter, where a smaller standard deviation indicates higher memory resolution.

The other distribution is a uniform distribution of recall errors. Since the target orientation is not remembered and the participant guessed, the recall error is not related to the target orientation, forming a uniform distribution (Bays, 2016; Bays et al., 2009; Zhang & Luck, 2008). The height of this uniform distribution provides the guess rate (*g*). By subtracting this rate from one (1-*g*), we derive the accessibility parameter, which represents the probability that an orientation was successfully retrieved. We estimated these parameters separately for each condition using the maximum likelihood estimation method in MemToolbox (Suchow et al., 2013) in MATLAB 2023b (The MathWorks, Inc., Natick, MA, USA).

#### Target Confusability Competition (TCC) model

The TCC model assumes that when recalling a target orientation, all possible orientation values in memory generate activation signals proportional to their similarity to the target. These activation signals are sampled from a Gaussian distribution with mean *d*_*x*_ and variance 1 (*m*_*x*_ ~ *N*(*d*_*x*_,1)). The mean memory signal for each orientation (*d*_*x*_) depends on two factors: its psychophysical similarity to the target orientation, captured by a similarity function *f(x)*, and overall memory strength, represented by the parameter *d′*. These are combined as *d*_*x*_ = *d′ f(x)*. The model then selects whichever orientation produces the highest activation signal as the response. Critically, the model has only one free parameter (*d′*), which reflects overall memory strength, while the similarity function *f(x)* is derived empirically. We assumed independent noise across different orientation values, following the original work (Schurgin et al., 2020).

For the similarity function *f(x)*, we faced a unique challenge: unlike colors, object orientations have physical endpoints that can introduce both circular (orientation-like) and bounded (location-like) similarity structures. To determine the most appropriate similarity function for our data, we analyzed publicly available psychophysical similarity functions from Tomic and Bays (2024), which include empirically and synthetically measured similarity profiles for multiple feature dimensions. We compared synthetic TCC similarity functions across a range of tuning widths against our empirical error distribution by computing mean squared error (MSE) for each candidate function. We also examined orientation-specific and location-specific similarity functions from the published data to assess whether our data reflected pure orientation memory or contained location-like characteristics. The best-fitting similarity function was selected based on minimum MSE and used for all subsequent TCC model fits.

#### Hypothesis testing

All statistical analyses were conducted in JASP (Version 0.95.2; JASP Team, 2025). In order to statistically compare the attention conditions, we ran a Bayesian paired-samples *t*-test. Given a prior estimation of a medium effect size, we employed a Cauchy prior with a scale parameter of 0.08 (Schmalz et al., 2023). In addition to these analyses, we also conducted classical frequentist tests, including a paired-samples *t*-test and a Wilcoxon signed-rank test when the assumption of normality was not met. Effect sizes for the paired-samples *t*-tests are reported as Cohen’s *d*_*z*_, calculated as the mean difference divided by the standard deviation of the difference scores (Lakens, 2013). This accounts for the within-subject correlation between measures.

### Results

#### Mean absolute error

**Fig. 2:**
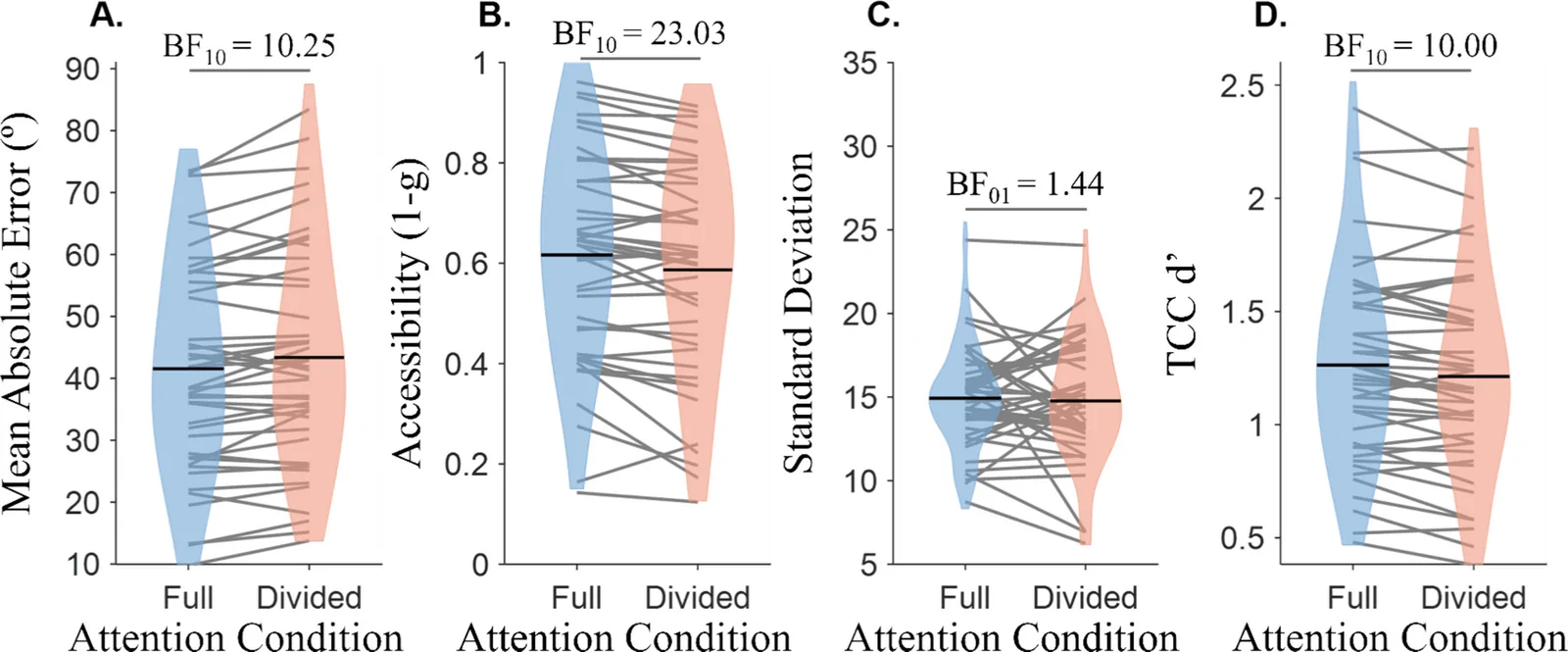
**A** Mean absolute error during full and divided attention conditions. Participants exhibited a significantly higher error rate during divided attention compared to full attention. (**B**) Accessibility (1 ˗ guess rate, estimated from the mixture model) of memories in full and divided attention conditions. Divided attention led to a higher guess rate, implying reduced accessibility of memories, suggesting reduced memory accessibility. (**C**) Standard deviation (1/precision, estimated from the mixture model) of memory representations across full and divided attention conditions. No significant difference in precision was observed between the two conditions. (**D**) Memory strength (*d′*, estimated from the TCC model) for full and divided attention conditions. Participants showed higher memory strength in the full-attention condition than in the divided-attention condition

To test whether divided attention impacts LTM retrieval, first, we calculated the absolute value of the degree difference between the memory orientation and response for each condition and participant separately. Then, we used a paired samples *t*-test to compare participants’ mean absolute error rate across full and divided attention conditions. The mean absolute error was higher during divided attention (*M* = 43.36) compared to full attention (*M* = 41.53; BF_10_ =10.25; *t* (43) = −3.40, *p* <.001, Cohen’s *d*_*z*_ = - 0.51, 95% CI [−0.825, −0.195]), suggesting that divided attention was detrimental for retrieval (Fig. 2A).

#### Accessibility

The accessibility (i.e., 1 ˗ guess rate) for each participant was estimated using mixture modeling. A paired-samples *t*-test was conducted to test whether divided attention affected the accessibility of the representations. Accessibility was lower in the divided attention (*M* = 0.58) condition compared to the full attention condition (*M* = 0.62, *SD* = 0.21; BF_10_ = 23.03; *t* (43) = −3.79, *p* <.001, Cohen’s *d*_*z*_ = 0.57, 95% CI [−0.888, −0.250] (Fig. 2B). Thus, divided attention disrupted the accessibility of memories.

#### Precision

Each participant’s standard deviation (1/precision) was obtained using mixture modeling. A paired-samples *t*-test was conducted to test whether divided attention affected the precision of the representations. There was anecdotal evidence in favor of the null hypothesis of no significant difference in standard deviation between the divided attention and full attention conditions (BF_01_ =1.44; *W* (43) = 510.00, *p* =.867, *RBC* = −0.03, 95% CI [−0.253, 0.338] (Fig. 2C), suggesting the precision of representations might not be affected by divided attention.

#### Memory strength

The memory strength (i.e., d*′* estimated from TCC model) for each participant was estimated by fitting a TCC model to the response offset separately for each attention condition. A paired-samples *t*-test was conducted to test whether divided attention affected the memory strength. Memory strength was lower in the divided attention (*M* = 1.213) condition compared to the full attention condition (*M* = 1.263; BF_10_ = 10.00; *t* (43) = 3.39, *p* =.002, Cohen’s *d*_*z*_ = 0.51, 95% CI [0.194, 0.823] (Fig. 2D).

#### Model comparison

To evaluate which computational framework best accounted for participants’ response distributions, we compared the mixture model and the TCC model using Akaike Information Criterion (AIC) and the Bayesian Information Criterion (BIC) and at the individual-subject level. Both AIC and BIC are metrics used for model comparison that reward the goodness of fit while penalizing the model for the number of parameters it uses. This ensures that a model is not preferred simply because it is more complex. Because these metrics represent a “loss” of information, lower values indicate a better balance between accuracy and simplicity. Across both attention conditions, the TCC model provided better fits. In the full-attention condition, the TCC model yielded lower AIC (*M* = 396.40) and BIC (*M* = 399.59) values compared to the mixture model (AIC: *M* = 1,851.09; BIC: *M* = 1,861.15), with TCC preferred for all 44 participants. Similarly, in the divided-attention condition, where the TCC model again produced lower AIC (*M* = 413.36) and BIC (*M* = 416.55) values relative to the mixture model (AIC: *M* = 1,869.73; BIC: *M* = 1,879.79), and was favored for all 44 participants. These results indicate that the TCC model offers a better account of the data across conditions.

#### Divided attention task accuracy and correlation analyses

**Fig. 3:**
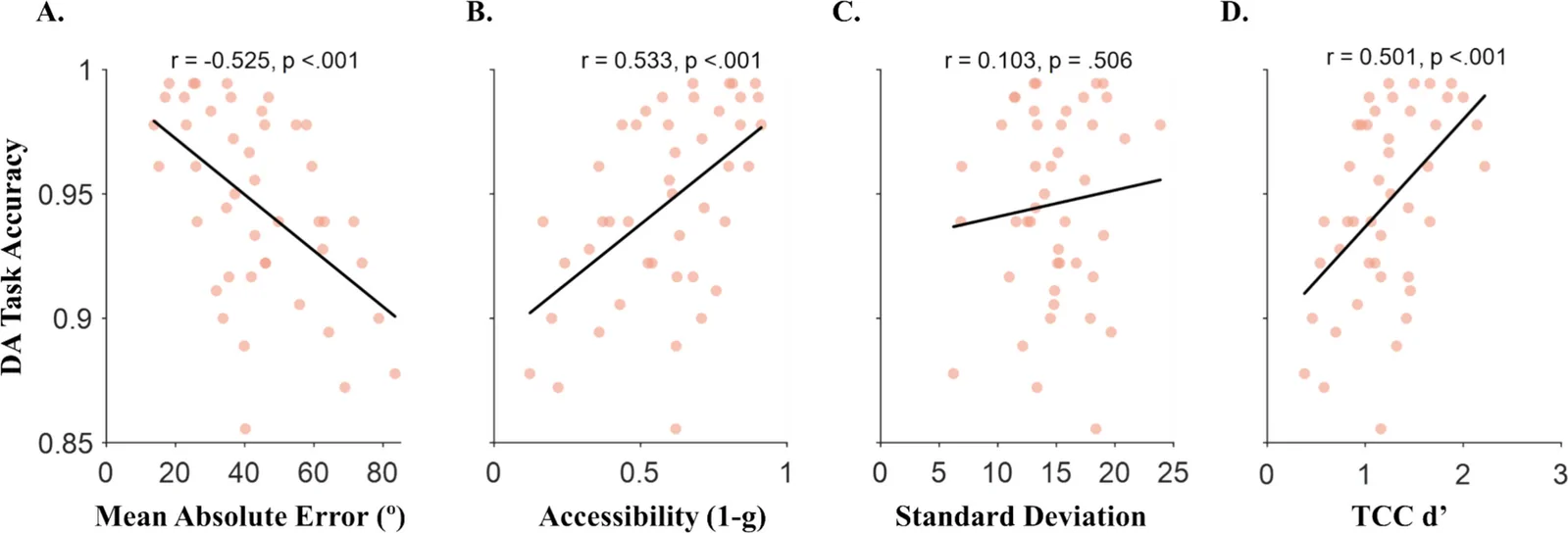
**A** Correlation between divided attention (DA) task accuracy and mean absolute error on divided attention trials. Higher DA task accuracy was associated with lower mean absolute error, indicating that better performance on the secondary task was linked to reduced memory accuracy. (**B**) Correlation between DA task accuracy and accessibility (1 ˗ guess rate). Higher DA task accuracy was associated with greater accessibility on divided attention trials, suggesting that participants who were more accurate on the secondary task were also more likely to retrieve stored memories. (**C**) Correlation between DA task accuracy and standard deviation (1/precision) of memory representations. No significant relationship was observed, indicating that memory precision on divided attention trials was unaffected by variation in secondary task performance. (**D**) Correlation between DA task accuracy and memory strength (TCC *d′*) on divided attention trials. Higher DA task accuracy was associated with greater memory strength, suggesting that participants who were more accurate on the secondary task were also more likely to have stronger memories

Participants demonstrated high performance on the divided attention task, with a mean accuracy of 0.942 (*SD* = 0.040), and scores ranging from 0.856 to 0.994. The divided attention task accuracy showed a significant negative correlation with mean absolute error (*r* = −0.525, *p* <.001), indicating that higher secondary task accuracy was linked to lower mean absolute error during divided attention. Similarly, divided attention task accuracy was significantly correlated with accessibility (*r* = 0.533, *p* <.001) and memory strength (*r* = 0.501, *p* <.001), with higher secondary task accuracy associated with greater accessibility and stronger memory during divided attention. No significant correlation was observed between divided attention task accuracy and precision (*r* = −0.103065, *p* =.67451). These findings suggest that participants did not trade off retrieval performance to maintain accuracy on the secondary task. Instead, better performance on both tasks likely reflects moments of stronger task engagement, indicating shared fluctuations in attention rather than a competition between memory and secondary task performance (Adam & deBettencourt, 2019; deBettencourt et al., 2016; Madore et al., 2020) (Fig. 3).

## Experiment 2

In Experiment 1, there was a potential confounding factor in the Divided Attention condition. Specifically, the divided attention condition required participants to search for a target among objects and also remember their response (whether the red star was on the left or right) while performing the memory retrieval task. This added an extra working memory load during retrieval under the divided attention condition, making it unclear whether the observed effects were due to the divided attention or the additional working memory load. We conducted a second experiment designed to eliminate this confound. In the second experiment, we adjusted the procedure so that participants responded to the divided attention task immediately after viewing the 12 objects and the red star. This change ensures that any effects observed in the divided attention condition are not due to the additional cognitive load of maintaining the search response during memory retrieval, thereby isolating the impact of processing the visual objects prior to retrieval. This modification aimed to provide clearer insights into the effects of divided attention on memory retrieval without the influence of cognitive load.

### Method

#### Participants

Fifty-four undergraduate students participated in the experiment for course credit. Participants who reported having normal or corrected-to-normal vision and having no neuropsychological disorders were accepted for the experiment. Seven participants’ data were removed from the analysis: Two participants did not complete the study, four participants’ mean absolute error exceeded the chance level (90˚), one participant’s parameter estimates were 2.5 standard deviations away from the grand average, and one participant had a below-chance accuracy for the divided attention task. Thus, the final analyses included 44 participants (35 female, nine male; *M*_age_ = 20.97 years). The study was approved by the institutional ethics committee.

#### Materials, design, and procedure

The materials and design were identical to Experiment 1. The procedure was also identical to Experiment 1, with the following exception: In the Divided Attention condition, participants were required to respond to the divided attention task immediately after seeing the task, before the retrieval response. In all other respects, including the sequence of encoding, filler task, retrieval cue presentation, and feedback mechanisms, the procedures remained the same as in Experiment 1.

### Results

#### Mean absolute error

**Fig. 4:**
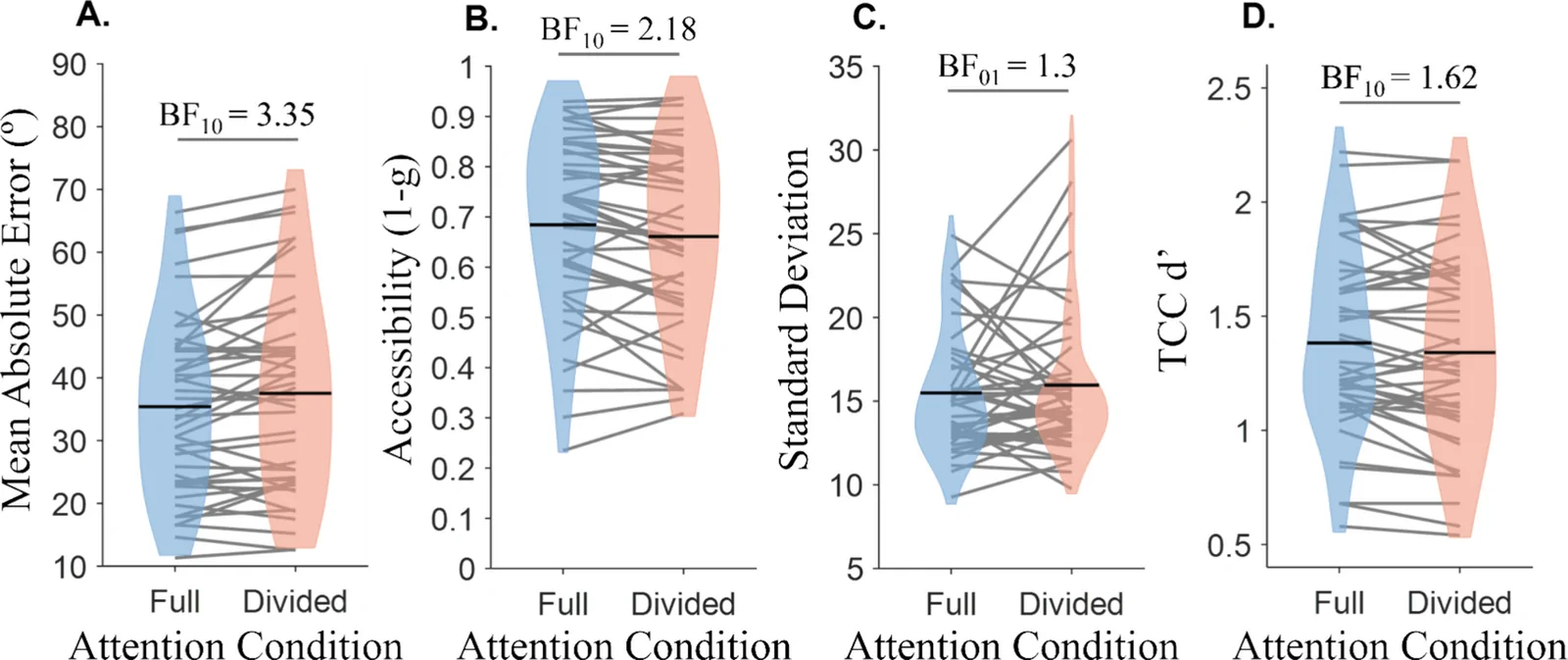
**A** Mean absolute error during full and divided attention conditions. Participants exhibited a significantly higher error rate during divided attention compared to full attention. (**B**) Accessibility (1 ˗ guess rate, estimated from the mixture model) of memories in full and divided attention conditions. Divided attention led to a higher guess rate, implying reduced accessibility of memories. (**C**) Standard deviation (1/precision, estimated from the mixture model) of memory representations across full and divided attention conditions. No significant difference in precision was observed between the two conditions. (**D**) Memory strength (*d′*, estimated from the TCC model) for full and divided attention conditions. Participants showed higher memory strength in the full-attention condition than in the divided-attention condition

To test whether divided attention impacts LTM retrieval, first, we calculated the absolute value of the degree difference between the memory orientation and response for each condition and participant separately. Then, we used a paired-samples *t*-test to compare participants’ mean absolute error rate across full and divided attention conditions. The mean absolute error was higher during divided attention (*M* = 37.53) compared to full attention (*M* = 35.40, BF_10_ =3.35; *t*(43) = 2.72, *p* = 0.009, Cohen’s *d*_*z*_ = 0.41, 95% CI [0.101, 0.716]), suggesting that divided attention was detrimental for retrieval (Fig. 4A).

#### Accessibility

The accessibility (i.e., 1 ˗ guess rate) for each participant was estimated using mixture modeling. A paired-samples *t*-test was conducted to test whether divided attention affected the accessibility of the representations. Accessibility was lower in the divided attention (*M* = 0.66) condition compared to the full attention condition (*M* = 0.68; BF_10_ = 2.18; *t* (43) = 2.38, *p* = 0.02, Cohen’s *d*_*z*_ = 0.37, 95% CI [−0.662, −0.052] (Fig. 4B). Thus, divided attention disrupted the accessibility of memories.

#### Precision

Each participant’s standard deviation (1/precision) was obtained using mixture modeling. A paired-samples *t*-test was conducted to test whether divided attention affected the precision of the representations. There was anecdotal evidence favoring the null hypothesis of no significant difference in standard deviation between the divided attention and full attention conditions (BF_01_ =1.30; *W* (43) = 453.0, *p* = 0.63, *RBC* = 0.085, 95% CI [−0.401, 0.249] (Fig. 4C), suggesting the precision of representations might not be affected by divided attention.

#### Memory strength

The memory strength (i.e., *d′* estimated from the TCC model) for each participant was estimated by fitting a TCC model to the response offset separately for each attention condition. A paired-samples t-test was conducted to test whether divided attention affected the memory strength. There was anecdotal evidence for lower memory strength in the divided attention condition (*M* = 1.34) compared to the full attention condition (*M* = 1.38; BF_10_ = 1.62; *t* (43) = 2.09, *p* =.043, Cohen’s *d*_*z*_ = 0.32, 95% CI [0.01, 0.616] (Fig. 4D).

#### Model comparison

To evaluate which computational framework best accounted for participants’ response distributions, we compared the mixture model and the TCC model using AIC and the BIC at the individual-subject level. Across both attention conditions, the TCC model provided better fits. In the full-attention condition, the TCC model yielded lower AIC (*M* = 350.8) and BIC (*M* = 354.0) values compared to the mixture model (AIC: *M* = 1,809; BIC: *M* = 1,819), with TCC preferred for all 44 participants. Similarly, in the divided-attention condition, where the TCC model again produced lower AIC (*M* =365.6) and BIC (*M* = 368.8) values relative to the mixture model (AIC: *M* = 1,824; BIC: *M* = 1,834), and was favored for all 44 participants. These results indicate that the TCC model offers a superior account of the data across conditions.

#### Divided attention task accuracy and correlation analyses

**Fig. 5:**
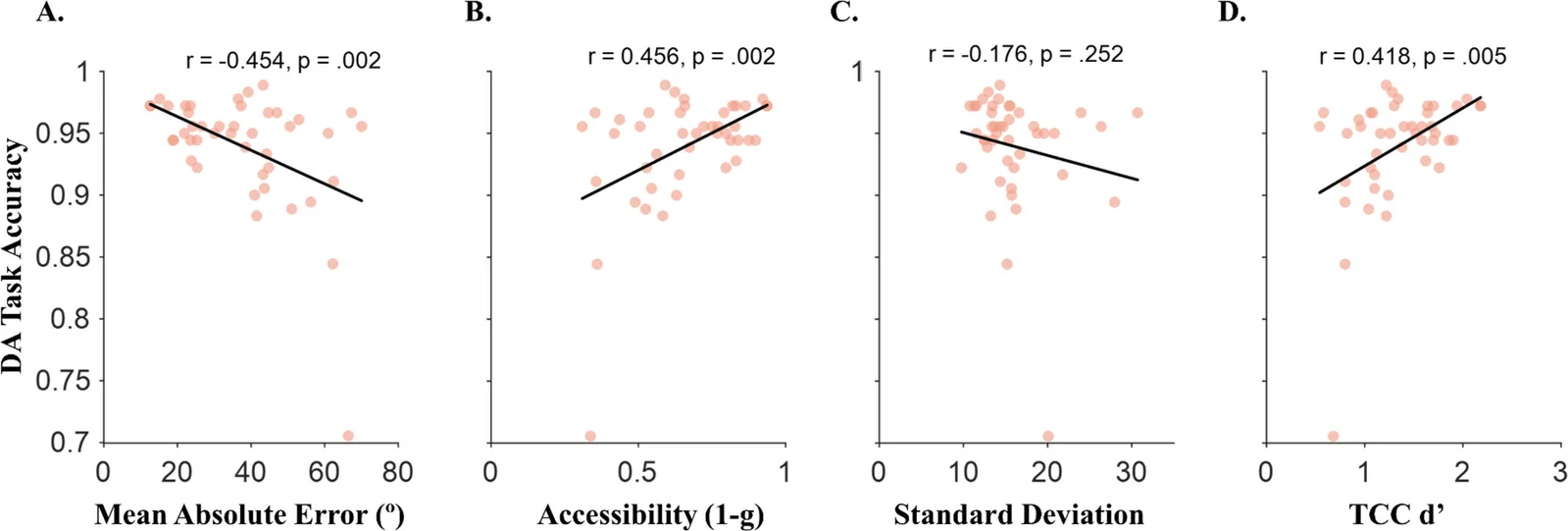
**A** Correlation between divided attention (DA) task accuracy and mean absolute error on divided attention trials. Higher DA task accuracy was associated with lower mean absolute error, indicating that better performance on the secondary task was linked to reduced memory accuracy. (**B**) Correlation between DA task accuracy and accessibility (1 ˗ guess rate) on divided attention trials. Higher DA task accuracy was associated with greater accessibility, suggesting that participants who were more accurate on the secondary task were also more likely to retrieve stored memories. (**C**) Correlation between DA task accuracy and standard deviation (1/precision) of memory representations on divided attention trials. No significant relationship was observed, indicating that memory precision was unaffected by variation in secondary task performance. (**D**) Correlation between DA task accuracy and memory strength (TCC *d′*) on divided attention trials. Higher DA task accuracy was associated with greater memory strength, suggesting that participants who were more accurate on the secondary task were also more likely to have stronger memories

Participants demonstrated high performance on the divided attention task, with a mean accuracy of 0.926 (*SD* = 0.102), and scores ranging from 0.71 to 0.989. Divided attention task accuracy showed a significant negative correlation with mean absolute error (*r* = −0.454, *p* = 0.002), indicating that higher task accuracy was linked to lower mean absolute error during divided attention. Similarly, divided attention task accuracy was significantly correlated with accessibility (*r* = 0.456, *p* = 0.002) and memory strength (*r* = 0.418, *p* =.005), with higher secondary task accuracy associated with greater accessibility and stronger memory during divided attention. No significant correlation was observed between divided attention task accuracy and precision (*r* = −0.176, *p* = 0.252). These results, consistent with those of Experiment 1, further support the conclusion that lower accessibility is not due to a tradeoff between the memory task and secondary task performance (Fig. 5).

#### Contribution of working memory load to the divided attention effect across experiments

To assess if working memory (WM) load component of Experiment 1 had any influence on the observed results, we ran independent-samples *t*-tests to directly compare the size of the divided attention effect (divided minus full attention) between Experiments 1 and 2 for each parameter of mixture model: accessibility precision, TCC model: *d′* and mean absolute error. For accessibility (1–g), the difference between experiments was not significant, t(86) = –0.50, p =.622, with anecdotal Bayesian evidence supporting the null (BF₀₁ = 1.28). For precision (SD), there was similarly no reliable difference, *t*(86) = 0.34, *p* =.730, BF₀₁ = 1.22. For TCC *d′*, the comparison again showed no significant difference, *t*(86) = 0.35, *p* =.730, BF₀₁ = 1.3. For mean absolute error, the comparison once again showed no significant difference, *t*(86) = 0.32, *p* =.748, BF₀₁ = 1.3. Overall, these results indicate that the divided attention effects were statistically comparable across experiments. While the effects appeared numerically smaller in Experiment 2, the analyses provide anecdotal evidence that this difference does not reflect a meaningful change in the impact of divided attention, suggesting that reduced working memory load did not reliably moderate the disruption in accessibility. However, it is important to note that the comparison between the two experiments was exploratory and should be interpreted with caution, as participants were not randomly assigned to the two experiments. Although the recruitment pools and procedures were consistent, factors such as the time of year or different experimenters could have influenced the results. Moreover, a post hoc power analysis revealed that our sample size (*N* = 88) provided 65% power to detect a medium effect size (Cohen’s *d* = 0.50) between experiments. While this suggests the comparison may be underpowered for smaller effects, the observed Bayesian evidence (BF₀₁ ∼ 1.3) consistently favored the null, providing weak support for the conclusion that the impact of divided attention did not differ between the two experiments.

## Discussion

In this study, we aimed to investigate how divided attention impacts accessibility and precision during LTM retrieval. Our findings suggest that divided attention disrupts retrieval, increasing errors made when participants recall the orientations of previously studied objects. Using a mixture-modeling approach, we found that divided attention specifically disrupted the accessibility of memoranda. Interestingly, the precision parameter remained unaffected, implying that divided attention did not reduce the precision of retrieved memories.

The explanation for the selective costs of divided attention on accessibility but not precision can be twofold. First, the automatic reactivation of memory traces, as proposed by the component process model, might have enabled participants to retrieve memories with adequate precision despite the presence of distractors. However, the limited attentional resources in the divided attention condition could have hindered the initiation and maintenance of the retrieval mode, leading to decreased accessibility. This finding aligns with the studies that show being in a retrieval mode or having a retrieval orientation increases memory accessibility (Alban & Annibal, 2022; Hornberger et al., 2006; Rugg & Wilding, 2000).

Second, the asymmetry between precision and accessibility can be explained by the competition between the retrieval and distractor tasks for the same buffer when they use overlapping representational modalities (e.g., visual, auditory, tactile). Previous studies have shown that divided attention has detrimental effects on LTM retrieval, particularly when the retrieval task and distractor task share the same representational system (e.g., words vs. digits; Fernandes & Moscovitch, 2000, 2002, 2003). This interpretation aligns with findings from WM literature, where domain-specific resources become susceptible to interference when two tasks or representations are in the same domain (Cocchini et al., 2002; Kane et al., 2004; Kovacs et al., 2019; Shah & Miyake, 1996). Consequently, when stored in the same buffer, retrieved memory items may become inaccessible rather than less precise under divided attention conditions. Since both tasks in our study used the same modality, this may indicate a domain-specific effect. However, future research is necessary to empirically determine whether these results reflect a material-specific or domain-general divided attention effect.

Despite numerically weaker effects in Experiment 2, comparisons of the size of the divided attention effect across experiments revealed no reliable differences in accessibility, precision, TCC *d′*, or mean absolute error, with anecdotal Bayesian evidence supporting the null. As discussed above, the null differences might simply be due to modest statistical power, but here we consider other potential accounts of the absence of additional cost of WM load. First, it is possible that divided attention during retrieval, rather than WM load, is sufficient to disrupt accessibility. Second, the WM load in Experiment 1 may not have strongly interfered with retrieval because it relied on a different representational format: the divided-attention task response could be maintained in a motor or verbal format, whereas memory retrieval relied on visuospatial representations. Third, it is possible that WM load costs occur early and briefly, such that storing the response only during the divided attention task (Experiment 2) or maintaining it for longer, until after the main retrieval response (Experiment 1), does not result in different levels of disruption. Future work that directly manipulates the modality, duration, and representational overlap of concurrent WM load during retrieval may provide clearer tests of the interplay between attention and memory on different timescales.

The lack of precision decrements under divided attention contradicts our hypothesis that posits divided attention mainly disrupts the precision, not accessibility, of memories. We developed this hypothesis based on the evidence suggesting that recall tasks are more susceptible to the costs of divided attention than recognition tasks (Craik et al., 2018; Fernandes & Moscovitch, 2002, 2003; but see Green et al., 2021). Accordingly, we proposed that larger divided attention costs in recall could reflect disruptions to precision, whereas low-precision but accessible memories might be sufficient for recognition.

However, we found the exact opposite pattern. Contrary to our hypothesis, the accessibility of representations suffered from divided attention, while the precision of the memories remained unaffected. Consequently, it remains unclear why the recall tasks are more prone to distraction than the recognition tasks. One possibility is that probes included in recognition tasks facilitate memory access despite attention being divided. Therefore, even if there is a decrease in accessibility due to divided attention, the probe in recognition tasks might counteract this effect by aiding memory access. Another possibility is that participants might use a different strategy in recall tasks than in recognition tasks, opting to store items with high precision but not necessarily all of them. This approach might allow participants to selectively learn or retrieve fewer items, preserving precision even under divided attention. Future studies are needed to test these possible remaining explanations.

The costs of divided attention were evident despite the retrieval cue being presented again at the test. This finding provides compelling evidence that the retrieval process requires re-encoding (Clos et al., 2019; Roediger & Butler, 2011; Roediger & Karpicke, 2006; Wang et al., 2019). Previously, Dudukovic et al. (2009) investigated the influence of attention during initial retrieval on subsequent item recognition and source memory tests. Their results showed that items initially tested under full attention were more likely to be remembered in follow-up tests than those initially subjected to divided attention. The presence of divided attention during initial retrieval reduced the chances of subsequent recognition even for items correctly recognized in the first test (but see Buchin & Mulligan, 2017, 2019; Mulligan & Picklesimer, 2016). Similarly, in our study, participants had two chances to retrieve the accurate orientation associated with the object. One was when the memory object was presented in upright orientation as the retrieval cue, and the other was at the test stage when participants were presented with the memory object in random orientation to rotate it and match the memorized orientation. Despite the second chance, participants could not reactivate the original, uninterrupted memory trace after experiencing divided attention, suggesting that the initially distorted memory might have replaced the original trace.

Divided attention can disrupt several stages of LTM retrieval (Ataseven et al., 2023). First, since the divided attention task was given right after the retrieval cue, it could have disrupted the processing of the cue or the search for the target LTM. This aligns with Naveh-Benjamin and Guez (2000), who argued that attentional selection during the cue-elaboration-search phase of LTM retrieval demands increased cognitive resources. Second, given that retrieved LTMs are suggested to be reactivated in WM (Favila et al., 2020; Fukuda & Woodman, 2017; Gisquet-Verrier & Riccio, 2012; Vo et al., 2022), the divided attention task could have interfered with the retrieved contents kept in WM. Specifically, during the test phase, the increased WM load due to the simultaneous storage of both the retrieved item and the search target might lead to the deterioration of these items, given the inverse relationship between WM load and storage abilities (Bays et al., 2009; Zhang & Luck, 2008). While there is growing evidence supporting the existence of multiple stages of LTM retrieval, how divided attention impacts each stage remains unclear (Ataseven et al., 2023). Future research that systematically isolates the impact of divided attention on each stage is needed to provide deeper insights into the role of attention in different LTM retrieval stages.

Several recent studies using continuous report paradigms in LTM have similarly reported selective effects on accessibility. For example, *the retrieval practice effect* is a well-established phenomenon showing that LTM retrieval improves performance in a subsequent memory test (e.g., Carpenter et al., 2009; Carrier & Pashler, 1992; Roediger & Karpicke, 2006; Rohrer et al., 2010). To better understand how retrieval practice impacts memory performance, Sutterer and Awh (2016) employed a continuous-report paradigm to dissociate the accessibility and precision of representations retrieved from LTM. They discovered that retrieval practice enhances the accessibility of representations while precision remains unchanged. In the context of forgetting, Berens et al. (2020) investigated how the precision and accessibility of representations in LTM change over time. Their results showed that forgetting was associated with decreased accessibility, while precision remained stable. In line with these studies, the current findings support a dissociation between accessibility and precision, showing that accessibility, not precision, is affected during LTM retrieval. Together, these studies imply that factors influencing LTM retrieval may disproportionately affect the likelihood of accessing stored information rather than the fidelity of the memory itself.

## Limitations and future directions

Recently, Schurgin et al. (2020) proposed the Target Confusability Competition (TCC) model, arguing that memory errors arise from a single underlying process – confusability-based memory strength (*d′*) – rather than separable constructs such as guessing and precision. This framework challenges mixture models by suggesting that an increase in guessing could sometimes reflect extremely imprecise, but not absent, memory representations. Here, the TCC model not only replicated our core finding that divided attention reduces memory performance but also provided consistently better fits than the mixture model in all conditions. While the TCC model provides a compelling account grounded in perceptual similarity, it does not fully explain the behavioral and neural dissociations observed in accessibility and precision (Bays et al., 2009; Berens et al., 2020; Brady et al., 2013; Fan & Turk-Browne, 2013; Richter et al., 2016; Zhang & Luck, 2008). Moreover, by using a single parameter, TCC offers a more parsimonious account of the data, whereas the mixture model provides critical complementary insight into *how* divided attention affects memory. Importantly, despite their differences, both models converge on the same conclusion that divided attention significantly reduces memory performance.

Although the mixture model offers a convenient decomposition of accessibility and precision, behavioral data alone cannot definitively separate extremely low-precision memories from true guesses (Van Den Berg et al., 2014; Schurgin et al., 2020). In this framework, both the guess-rate parameter (reflecting the proportion of responses drawn from a uniform distribution) and the precision parameter (the concentration of the target-centered Von Mises distribution) are jointly estimated from the same continuous error distribution. The model thus assumes that each trial arises from one of these latent components and treats accessibility and precision as independent parameters by assumption, even though, in reality, weak or degraded traces could simultaneously reduce the likelihood of retrieval and its fidelity. Moreover, parameter estimates can be correlated and sensitive to sample size, potentially limiting power to detect small precision effects (Bays et al., 2009; Van Den Berg et al., 2014). It therefore remains possible that part of the observed increase in guess rate under divided attention reflects the retrieval of very coarse or degraded representations that fall below our precision threshold. Future work employing larger samples or neural measures may help determine whether divided attention truly spares precision or merely shifts the fidelity continuum toward lower resolution.

One might argue that the memory system involved in our design is not LTM, but WM, since we tested a relatively small number of items after a short delay period. However, we have taken deliberate steps to encourage LTM storage. First, by using a filler task, we aimed to prevent rehearsal typically associated with WM (Cowan, 2008; Jonides et al., 2008; Ranganath & Blumenfeld, 2005). Furthermore, by serially presenting six items during encoding, we aimed to exceed the typical capacity of visual WM, which is about 3 units of information (Adam et al., 2017; Awh et al., 2007; Cowan, 2010; Luck & Vogel, 2013; McCollough et al., 2007), resulting in the engagement of the long-term storage. Given that each item is tested after all six items have been studied, actively storing each item while reporting others is challenging. Thus, we argue that active rehearsal of studied items until the test is extremely unlikely. Nevertheless, we acknowledge that our design did not allow for consolidation of memory items through sleep, which is suggested to strengthen synaptic connections (Diekelmann & Born, 2010; Stickgold & Walker, 2007). Thus, future studies should explore the potential differences in the effects of divided attention on the precision and accessibility of memories before and after sleep.

## Constraints on generality

The present findings should be interpreted within several boundary conditions. First, the secondary task used here involved visuo-spatial search and maintenance demands. The goal of designing such a divided attention paradigm was to maximize otherwise subtle effects of divided attention. Therefore, it is important to note that the current findings are restricted to recall of visual associations under visual divided attention. It is currently unclear if the results generalize to verbal, semantic, or autobiographical memories, which may rely on distinct attentional and representational mechanisms and in divided attention tasks that tax verbal, semantic, or executive functions. Second, current experiments tested visual object–orientation associations over short delays, and it remains to be tested whether the impairment by divided attention and the dissociation between accessibility and precision are observable at longer time scales (e.g., during consolidation through sleep). Third, our sample consisted of healthy young adults, and individual differences in factors such as age, working memory capacity, or attentional control could modulate the extent to which divided attention disrupts accessibility and/or precision. Finally, our study was powered to detect medium-sized within-subject effects, and although Bayesian analyses provided anecdotal evidence for the null for precision, we cannot rule out the possibility that the precision parameter requires more power to detect subtle differences. Future research using larger samples, longer retention intervals, or neural measures of representational fidelity could more decisively test whether divided attention truly spares precision or merely attenuates it.

## Conclusion

Across two experiments, we showed that divided attention during LTM retrieval increases memory errors. Using a continuous-report paradigm combined with mixture modeling, we found that this effect was characterized by an increased rate of guess-like responses under divided attention, while the precision of responses remained unchanged. Although the Target Confusability Competition framework provided a better fit to the data, the observed pattern is also consistent with a reduction in memory accessibility rather than precision. Across models, the results consistently show that divided attention impairs successful memory retrieval. Moreover, our results suggest that this cost is likely irreversible, as it was observed despite the same retrieval cue being presented again after attention was divided. Together, these findings reveal the importance of attention for accessing previously acquired information.

## Data Availability

The data and materials for all experiments of the current study are available in a public Open Science Framework repository: https://osf.io/yzw7c/

## Code availability

The code to create and analyze all experiments of the current study is available in a public Open Science Framework repository: https://osf.io/yzw7c/

## Declarations

### Open Practices Statement

None of the experiments were preregistered.
